# Intelligent decision support algorithm for distribution system restoration

**DOI:** 10.1186/s40064-016-2810-4

**Published:** 2016-07-26

**Authors:** Reetu Singh, Shabana Mehfuz, Parmod Kumar

**Affiliations:** 1 Jamia Millia Islamia, New Delhi, Delhi India; 2 Department of Electrical Engineering, Jamia Millia Islamia, New Delhi, Delhi India; 3 IRD, Maharaja Agrasen Institute of Technology, New Delhi, Delhi India

**Keywords:** Distribution system, Fuzzy logic, Grey system theory, Power system restoration, Transformer loading

## Abstract

Distribution system is the means of revenue for electric utility. It needs to be restored at the earliest if any feeder or complete system is tripped out due to fault or any other cause. Further, uncertainty of the loads, result in variations in the distribution network’s parameters. Thus, an intelligent algorithm incorporating hybrid fuzzy-grey relation, which can take into account the uncertainties and compare the sequences is discussed to analyse and restore the distribution system. The simulation studies are carried out to show the utility of the method by ranking the restoration plans for a typical distribution system. This algorithm also meets the smart grid requirements in terms of an automated restoration plan for the partial/full blackout of network.

## Background

 India’s blackout as two separate events on 30th and 31st July 2012, has forced the system design engineers to plan the restoration schemes which are more effective and efficient and takes into account the uncertainties involved in the transmission distribution network. Power system blackouts cannot be avoided. Their occurrence is rare, but their impact can be very serious. Further, the electric utilities also have loss of revenue. It is therefore essential to study, analyse and prepare the restoration plans in the event of tripping of a feeder or transmission line due to fault or other reasons (Fukuyama and Chiang 1995; Hsu et al. 1992; Hsiao and Chien 2000; Hsu and Kuo 1994; Lee et al. 1998; Ma et al. 1992). Some of the features of effective strategy plans are:

- The minimum number of consumers should be affected due to outage of the feeder.
- Power loss should be minimum on restoration of supply after blackout/feeder tripping.
- The structure of the radial network needs to be maintained as far as possible.
- The capacity of transformer, feeders, laterals and other network circuits should maintain and not exceed their capacity limits after restoration of the distribution network system.
- The critical consumers and loads should be given priority during strategy planning.
- For fast and techno economical solutions, there shall be ranking of the restoration plans in order to be followed by system operator.

The outage of feeder needs to be isolated to restore the maximum possible consumer’s load by restructuring the distribution network meeting the operational constraints and limits of feeder and equipments. In the present paper the criteria for selecting the restoration strategic plans are:

1. Minimizing the operation of switches.
2. Availability of supply to maximum consumer’s load, and
3. Meeting the constraints and unconstrained criteria.

Tsai et al. (1993), Berdandon et al. (2011), Feltes et al. (2006), Liu and Lin (2012), Chen et al. (2001); Lotfifard et al. (2011), Lim et al. (2006) and Nagata and Sasaki (2002) present the various methods for reconfiguration of networks after blackout or feeder outage. However, a limited work is available based on fuzzy-grey relation ranking method. Further, in most of these research works, constraint related to distribution transformer has not been included. Transformer is an important source of supply and hence we have incorporated the constraint of transformer capacity limit besides the constraints related to distribution network, like feeders and laterals capacity limitations. In order to compare and verify the accuracy of the study, a prototype distribution network of same structure (Chen et al. 2005) has been considered.

## Problem definition

The objective of present study is to minimize the number of switching operations for restoration of power supply to all consumer’s load and meeting the distribution system network constraints:

1. Let *λ*_1_ (s) be the minimum number of switching operations. Let N_sw_ be the maximum number of switches in the network. X is the position of switch during operartion. If a switch is opened from closed position or vice versa, the value of switch vector will be 1 and if there is no change in the status of switch, its value will be 0.
2. The maximum feeder’s loading *λ*_2_ (s) shall be within the rating of current capacity limitation, if *λ*_2_ (s) ≤ maximum (feeder lines loading). Similarly the maximum lateral loading, *λ*_3_ (s) is given by *λ*_3_ (s) ≤ maximum (lateral lines loading).
3. Restoration of maximum loads at faulted area.
4. Minimum switching operations in the restoration plan.
5. The load in feeder, laterals and transformers should be balanced as far as possible, and the overloading of electrical equipment should be avoided.
6. The reconfigured distribution system should be nearly close to the original system, and the radial structure of distribution system should be maintained.

## Mathematical model for reconfiguration

In order to design and develop the strategic plans for reconfiguration and implement them effectively, the following model of constraints and equalities are presented:

### Equalities/objective functions

#### Number of switching operations

If *λ*_1_ (S) defines the operations of the number of switches,

1 $$\lambda_{1} (s) = \sum\limits_{i = 1}^{{N_{sw} }} {X_{i} }$$

then, here, X_i_ is switch state vector given by [S_1_, S_2_, S_3_ …, S_NSW_], N_sw_ = The total switches that can be operated in the network under consideration, X_i_ = status of the switch. The conditions for the switch status are: X_i_ = 1, if switch is opened from closed position or vice versa, X_i_ = 0, if status of switch is not changed. Minimum number of switching operations indicates that the system will be more stability.

#### Maximum loading among backup feeders

The maximum loading, *λ*_2_ (S) among supported feeder is given by Eq. (2):

2 $$\lambda_{2} (S) = Max(I_{{FD_{i} }} ),\quad i = 1,2, \ldots ,N_{FD}$$

${\text{I}}_{{{\text{FD}}_{\text{i}} }}$ represents the current over the supported feeder FD_i_ after switching operations. N_FD_ defines the number of supported feeders. To meet the constraints criteria, λ_2_ (s) shall be minimised. This objective function will give the most loaded backup feeder and by this we can have the remaining marginal load.

#### Maximum loading among backup laterals

Like loading criteria for feeders the supported laterals shall also meet the load criteria. This objective function will give the most loaded backup laterals. A lesser value of λ_3_ (s) is preferred.

λ_3_ (s) is the capacity of supported laterals and LAT_i_ is the load current over the laterals after switching operation and N_*LAT*_ is the number of lateral branches. For techno-economic operation the λ_3_ (S), Eq. (3) is desired to be minimized:

3 $${\lambda _3}(S) = Max({I_{LA{T_i}}}),\quad i = 1,2, \ldots ,{N_{LAT}}$$

where λ_3_ (S) defines the supported laterals for maximum loading and $I_{{LAT_{i} }}$ defines current over of the supported lateral LAT_i_ after switching operation. N_LAT_ defines the number of laterals in the distribution network. The load on the laterals should be minimum for the best operating conditions during restoration.

#### Unbalanced loading of feeders

The feeders as well as laterals shall have the balanced loading of feeders and laterals. It is an important feature for line loss reduction and voltage stability criteria. Thus, the load unbalancing index of feeders and laterals can be computed using Eqs. (4) and (5) respectively.

4 $$\lambda_{4} (S) = \sqrt {\sum\limits_{i = 1}^{{N_{FD} }} {(LV_{{FD_{i} }} - LV_{FD} )^{2} } }$$

where, ${\text{LV}}_{{{\text{FD}}_{\text{i}} }}$ is percentage load level of feeder FD_i_ and LV_FD_ is percentage refrence load level which is given by Eq. (5)

5 $$LV_{FD} = \frac{{\sum\nolimits_{i = 1}^{{N_{FD} }} {I_{{FD_{i} }} } }}{{\sum\nolimits_{i = 1}^{{N_{FD} }} {IR_{{FD_{i} }} } }}*100$$

In the above equation ${\text{I}}_{{{\text{FD}}_{\text{i}} }}$ and ${\text{IR}}_{{{\text{FD}}_{\text{i}} }}$ represents the load current and rated load current of feeder. In order to improve the performance of the system the unbalancing loading index shall be as minimised.

#### Unbalanced loading of laterals

Similarly, the lateral branches unbalance load index λ_5_ (s) can be computed using equation:

6 $$\lambda_{5} (S) = \sqrt {\sum\limits_{i = 1}^{{N_{LAT} }} {(LV_{{LAT_{i} }} - LV_{LAT} )^{2} } }$$

where, ${\text{LV}}_{{{\text{LAT}}_{\text{i}} }}$ is percentage load level of lateral LAT_i_ and LV_LAT_ is percentage reference load level which is given by Eq. (7) as:

7 $$LV_{LAT} = \frac{{\sum\nolimits_{i = 1}^{{N_{LAT} }} {I_{{LAT_{i} }} } }}{{\sum\nolimits_{{}}^{{}} {IR_{{LAT_{i} }} } }}*100$$

In the above equation, ${\text{I}}_{{{\text{LAT}}_{\text{i}} }}$_and_${\text{IR}}_{{{\text{LAT}}_{\text{i}} }}$ represents the load current and rated load current of lateral respectively. This objective function is used to determine the degree of unbalanced loading of laterals, therefore, less value of λ_5_ (s) is preferred.

#### Maximum loading among backup transformer

Transformer is the main source of power supply to feeders and laterals. Its maximum loading capacity and unbalanced loading index after the isolation of the fault needs to be computed and checked. These shall be as minimum as possible. The minimization of maximum loading of transformer due to supported feeders and laterals is desirable. Maximum loading of transformer, λ_6_ (s) is computed by Eq. (8) as:

8 $$\lambda_{6} (S) = Max(I_{{TRS_{i} }} ),\quad i = 1,2, \ldots ,N_{TRS}$$

#### Unbalanced loading of transformer

The unbalanced loading index of transformer, λ_7_ (s) is given by Eq. (9), where,

9 $$\lambda_{7} (s) = \sqrt {\sum\limits_{i = 1}^{{N_{TRS} }} {(LV_{{TRS_{i} }} - LV_{TRS} )^{2} } }$$

where, LV_TRSi_ is percentage load level of transformer TRS_i_ and LV_TRS_ is percentage reference load level which is given by Eq. (10)

10 $$LV_{TRS} = \frac{{\sum\nolimits_{i = 1}^{{N_{TRS} }} {I_{{TRS_{i} }} } }}{{\sum\nolimits_{i = 1}^{{N_{TRS} }} {IR_{{TRS_{i} }} } }}*100$$

In the above equation ${\text{I}}_{{{\text{TRS}}_{\text{i}} }}$_and_${\text{IR}}_{{{\text{TRS}}_{\text{i}} }}$ represents the load current and rated load current of transformer respectively. This gives the degree of unbalance loading of transformer for the backup and the value of this function should be minimum.

### Constraints

To further optimize the switching operation for the reconfiguration of distribution system, the following constraints shall have to be met:

1. Open switch operation have been complemented by closed switch operation.
2. I_LATmin_ < I_j_ < I_LATmax_
3. I_FEEDERmin_ < I_j_ < I_FEEDERmax_
4. I_TRSmin_ < I_j_ < I_TRSmax_

## Fuzzy grey method

### Fuzzy multi criteria evolution

**Fig. 1:**
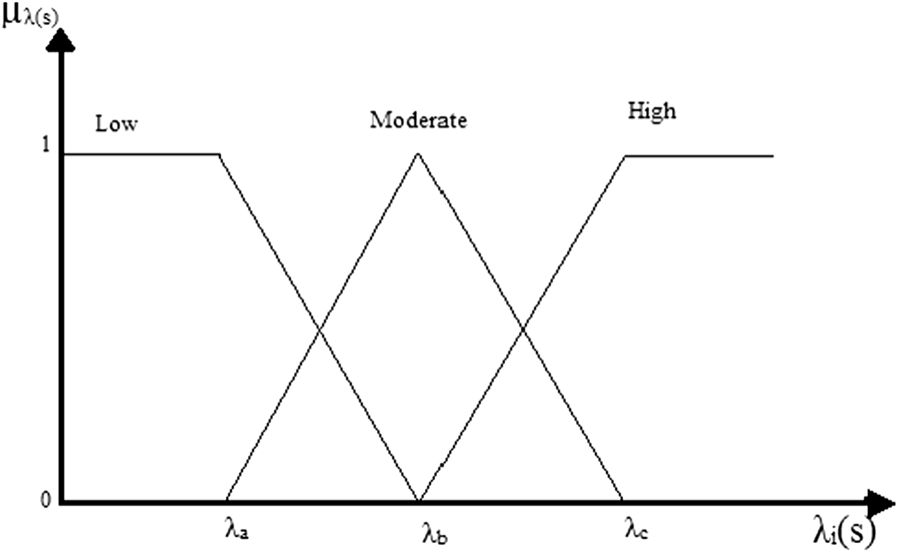
Triangular fuzzy function

The fuzzy logic method is a mathematical tool to make decision for vague and imprecise information in power system restoration problems (Chang 2008; Farahani et al. 2007; Gomes Flavio 2006; Gonzalez et al. 2012; Savier and Das 2007; Nagata et al. 1995; Pham et al. 2009; Wong and Lai 2000). Using fuzzy logic data base rules, a strategy with lesser switching operation and better load balance is achievable. Here, the linguistic terms like lesser, better etc. convey the vague nature of information. The restoration plan is considered more preferable if it involves fewer switching operations and better load balance. In restoration process, uncertainties arises when the feeder, lateral or transformer current is changing during the restoration process. These uncertainties can be taken into account using the fuzzy logic tool. It is based on rule-base (system operator experience), membership function of variable, and inference decision engine (IF THEN statement). One can consider the membership function of any type (triangular, sigmoid etc) but generally the fuzzy function is selected based on the nature of the problem. In the present study, three level (Low, Moderate and High) triangular fuzzy functions are considered to simplify the calculations during the restoration process, as shown in Fig. 1. “Appendix 2” presents the values of fuzzy membership function for different objective function. The fuzzy function transforms crisp value to fuzzy value which lie in the range {0, 1}. Then using rule-base and fuzzy inference decision procedure, the fuzzy value related to each defined variable/objective function is computed. These output fuzzy values, after inference, are transferred back to crisp values using de-fuzzification methods (either centre of gravity method or centre of area method). In the consequents the fuzzy sets are low, moderate and high, and can be crisply defined as 1, 0.5 and 0 respectively. Further inference is drawn by calculating the real value of objective function. By real value we can get the firing strength in IF-THEN rule and the weighted average. By using Eq. (11), the crisp de-fuzzification value is derived.

11 $$f_{i}^{*} = \frac{{\sum\nolimits_{j = 1}^{{N_{R} }} {\mu_{j} \times y_{j} } }}{{\sum\nolimits_{j = 1}^{{N_{R} }} {\mu_{j} } }}$$

where, µ_j_ and *y*_j_ are firing strengths of anticedents and consequences; N_R_ represents the number of fuzzy rules. The value of *f*_i_* represent the fitness degree of objective function ‘*f*_*i*_’ for each restoration plan. For example, the number of switching operations performed for the restoration plan 5 is 5. The rules are as follows:

- R1: IF *λ*_1_ (S) is low, THEN the plan is good.
- R2: IF *λ*_1_ (S) is moderate, THEN the plan is moderate.
- R3: IF *λ*_1_ (S) is high, THEN the plan is bad

The rule strength of R1, R2 and R3 will be 0.5, 0.5 and 0 respectively for the plan 5. After computing the rules, we use Eq. (11) to translate the rule results into real value by weighted average method. The singleton value for good, moderate and bad are 1, 0.5 and 0 respectively. The corresponding de-fuzzification value will be (0.5*1+0.5*0.5+0*0)/(0.5+0.5) = 0.750.

### Grey regression method

Grey relation theory is based upon the concept that available information is incomplete and/or unknown. It is data analysis technique to solve the multicriteria decision making (MCDM). Such problems (MCDM) are difficult to solve using fuzzy logic tools (Wong and Lai 2000; Zhang and Zhengeai 2008; Cheng et al. 1998; Deng 1982; Dong et al. 2003; Huang and Huang 1996; Liu and Forrest 2007; Deng 1989; Song et al. 2002; Tsai et al. 2003; Wong and Lai 1999; Chang and Yeh 2005; Huang et al. 2008; Lin et al. 2008). The coefficient of grey relation (GRC) of x_i_ with respect to x_0_ for kth term is given in Chen et al. (2005).

12 $$\gamma \left( {x_{0} \left( k \right), \, x_{i} \left( k \right)} \right) \, = \, \left\{ {\Delta_{\hbox{max} } - \Delta_{{{\text{o}}i}} \left( k \right) \, } \right\}/\left\{ {\Delta_{\hbox{max} } - \, \Delta_{\hbox{min} } } \right\}$$

where, *x*_0_ = (*x*_0_(1), *x*_0_(2), *x*_0_(3), …, *x*_0_(n)), o = 1, 2, 3, …, n, and *x*_*i*_ = (*x*_*i*_(1), *x*_*i*_(2), *x*_i_(3), …, *x*_*i*_(m)), i = 1, 2, 3,…, m.

$${\Delta _{{\rm{max}}}} = {{Max}}{\mkern 1mu} \left( {{x_0}\left( k \right) - {x_i}\left( k \right)} \right)$$

$${\Delta _{{\rm{min}}}} = {{ Min}}{\mkern 1mu} \left( {{x_0}\left( k \right) - {x_i}\left( k \right)} \right)$$

$$\Delta_{{{\text{o}}i}} \left( k \right) = \left| {\left( {x_{0} \left( k \right) - x_{i} \left( k \right)} \right)} \right|$$

The GRG between each comparative sequence *x*_i_ and refrence sequence *x*o is derived from average value of GRC. The order of relation between comparative and reference sequences is given by $\varGamma 0i$. Higher value of $\varGamma 0i$ means that the comparative sequence is more close to reference sequence than comparative sequence.

13 $$\varGamma_{0i} = \sum\limits_{k = 1}^{n} {\frac{1}{n}} \gamma (x_{0} (k),x_{i} (k))$$

In the next stage of the grey analysis, the GRA is used to measure the preference degree for all feasible restoration plans. The various steps for the fuzzy grey approach for ranking the restoration plans and selecting the satisfactory plan is presented in the fuzzy grey relation.

### Fuzzy-grey relation

In order to overcome the limitations of decision conditions related to grey relational method and fuzzy logic tool limitations, the two decision making tools with incomplete and vague information are fused together to form a hybrid fuzzy-grey relational tool (Lin et al. 2008; Basu and George 2014; Pereira Junior et al. 2014; Shahsavari et al. 2014; Liu et al. 2015). This tool overcomes the limitations in the two methods, and make the decision making more relevant and effective. Based on the minimum value of λ, the optimized objective function is decided among various alternatives. Optimized λ value is the best alternative switching operation sequence, loading on feeders, laterals and transformer. It provides the optimized solutions for decision making considering the various constraints and equalities. Choice of restoration plan is a type of multi criteria decision making problem which depends on all the objective functions and constraints considered. In this work we have tried to construct a measurement model via grey relational analysis to provide useful information and help system operator to make a right decision on the problem of service restoration.

**Fig. 2:**
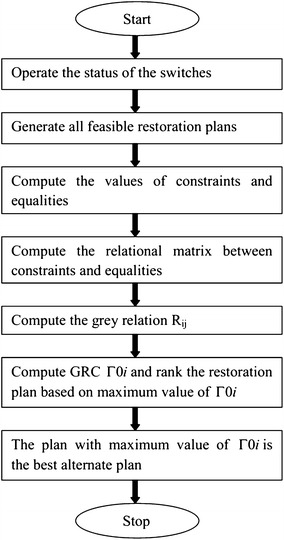
Flow chart for the restoration plan

Figure 2 represents the flow chart for the entire restoration algorithm. Starting with the on-off status of the switches, the feasible restoration plans are generated. Objective function values are computed using Eqs. (1)–(10) for the feasible plans. Further fitness degree of the objective function is evaluated using the fuzzy multicriteria evaluation method. The grey regression analysis (GRA) method is used to calculate the preference index of the restoration plans. Based on the grey regression grades (GRG) the plans are ranked according to their preference order. The addition of objective function’s minimization of unbalanced loading of transformer gives the stable restoration plans for the considered network. This improves the system reliability and stability, leading to the improved performance of the system.

## Numerical application

In order to show the utility of fuzzy-grey relation method for reconfiguration of distribution network, a distributed transformer as a source of supply is added to the network configuration (Chen et al. 2005).

**Fig. 3:**
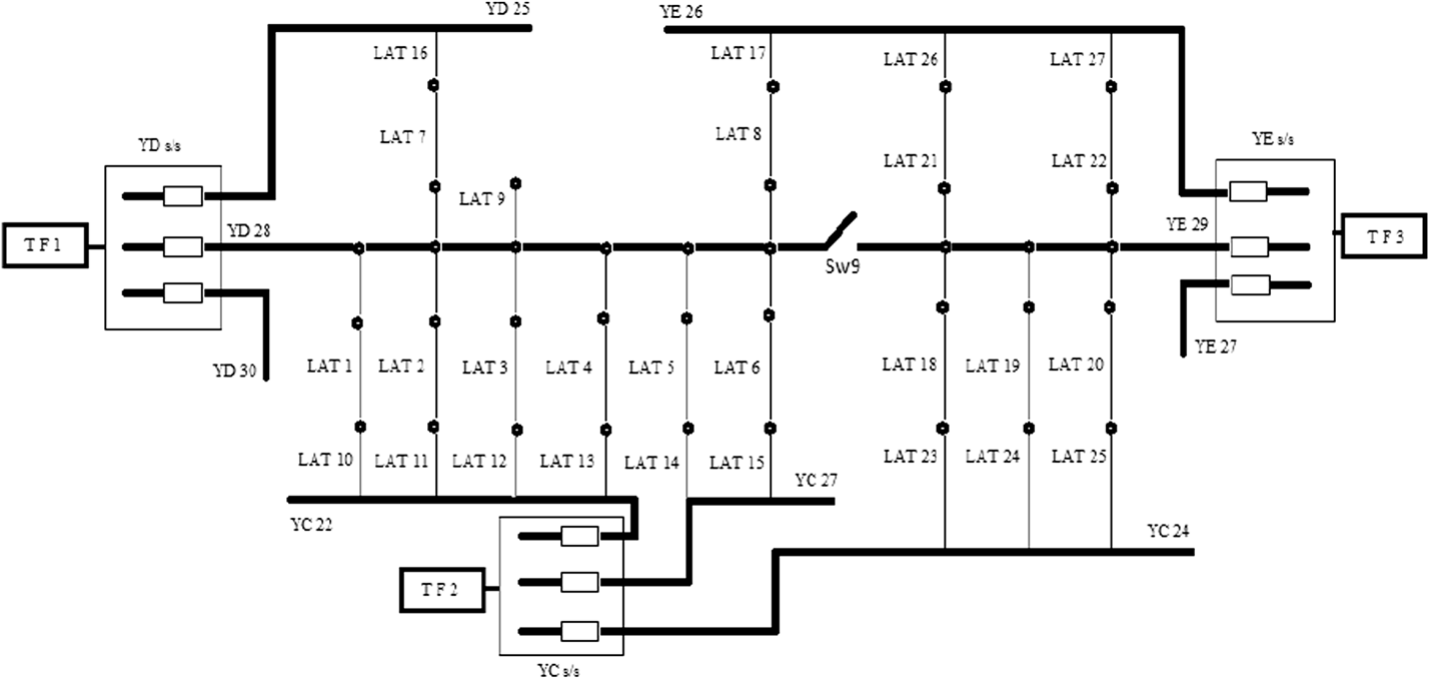
Distribution system

**Fig. 4:**
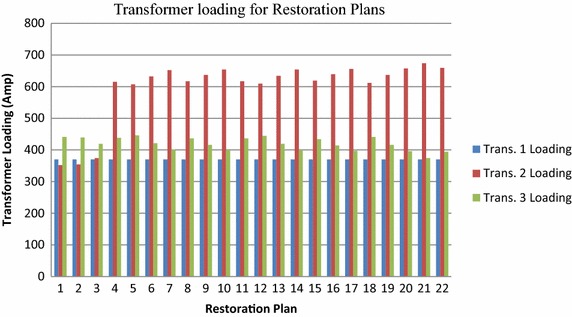
Transformer loading

**Fig. 5:**
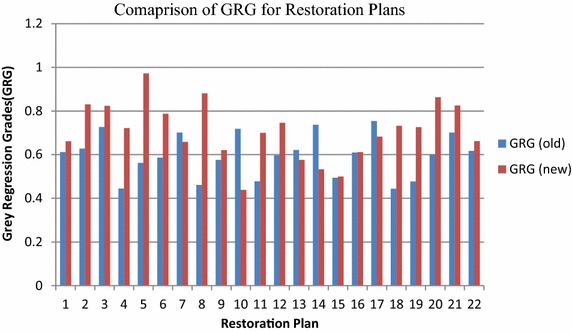
Result comparison

The distribution system of Taiwan Power Company is considered and presented in Fig. 3, which has main feeders YD_28_ supplying power to LAT1, LAT2, LAT3, LAT4, LAT5, LAT6, LAT7, LAT8 and LAT9. Each lateral has its supporting lateral (LAT10, LAT11, LAT12, LAT13, LAT14, LAT15, LAT16 and LAT17). These alternating laterals are connected to the main lateral with the help of switches. When a fault occurs in the system the switches operate to restore the out of service area. “Appendix 1” presents the pre-fault load current of feeders. “Appendix 2” presents the maximum capacity of each feeder, laterals, and the loading of transformer. The switch state vector, X comprises of the main switches S_W_ i (i = 1–9) and alternative switches S_W_ j (j = 1–8). Switch Sw9 always remain closed since lateral 9 is not connected with any other supporting lateral. To maintain the radial structure of the network, the switch open operation should be followed by switch close operation or vice versa. The switch pair for each restoration plan is given in Table 1 against each restoration plan based on the switching operation performed. The lateral, feeder and transformer loading is presented against the switching vector. All load values are in ampere. The maximum number of switching operations can be 8, thus, the total number of possible restoration plans are 2^8^ = 256. The rated capacity of feeder, lateral, and transformer are assumed as 450, 100, and 800 A respectively before the fault condition. Table 1 lists all the feasible restoration plans and the load currents on supporting lines after the restoration of supply. The 22 feasible restoration plans are selected from 256 possible restoration plans. The maximum switching operations possible are, therefore, 7 for the computations as per the switch state vector. Table 2 gives the values of all the objective functions. The corresponding data shown in Table 3 are obtained by fuzzy multi-criteria evaluation discussed in “Mathematical model for reconfiguration” section. This table gives us the values of reference sequence which is used further to calculate GRGs. the reference sequence selected for analysis is maximum value of fuzzy evaluation of objective functions: X_0_ = (0.750, 0.2522, 0.3469, 0.719, 0.391, 0.777, 0.625). The GRG values computed using Eq. (13) are presented in Table 4. The grey relation grades computed by Chen et al. (2005) are reproduced also in this table. After the GRA steps are completed, the preference ranking of feasible plans are derived with the related analysis and the ranking is presented in Table 5. From the table we can see that the plan 5 is having the highest rank. The plans get their ranking modified because of the addition of transformer objective functions. The loading of all the transformer present in the distribution system is graphically presented in Fig. 4. Additional objective function increases the stability of the system as the unbalancing of the transformer load can also be handled. The new plans are compared with the earlier ones in Fig. 5. Higher the number of objective functions more is the stability of the system as more parameters are considered. The difference of old and new GRG is calculated in Table 4. Further, mean of difference of new and old GRG is calculated, which comes out to be 0.1092. This means that the system performance has increased by 1.09 % with new GRG. The restoration plans are ranked according to the new GRG values. If the current exceeds in a particular element, it could fail and the current would be shunted to other network element which eventually may fail also. Here all equipments are considered so if feeder gets overloaded, it can transfer load to lateral and it can shift the load to the transformer and system gets more stable. Entire restoration algorithm consists of the fuzzy evaluation and fuzzy grey multicriteria given by flow chart. The inclusion of transformer loading parameters in fuzzy analysis makes the system more stable as more parameters are restored. In previous work, feeder and laterals were restored but in this work, transformer is included and restored after the fault which increases the stability and reliability of the system.

**Table 1:** Load current of supporting components after restoration

Feasible plan	Switch pair vector	L 10	L11	L12	L13	L14	L15	F_YD_	F_YC_	F_YE_	Tf1	Tf2	Tf3
1	11100000	76	84	86	37	34	31	441	352	260	370	352	441
2	10110000	76	39	86	84	34	31	439	354	260	370	354	439
3	01110000	51	84	86	84	34	31	419	374	260	370	374	419
4	11001000	76	84	24	37	99	31	438	290	325	370	615	438
5	00101000	51	39	86	37	99	31	446	282	325	370	607	446
6	10101000	76	39	86	37	99	31	421	307	325	370	632	421
7	01101000	51	84	86	37	99	31	401	327	325	370	652	401
8	10011000	76	39	24	84	99	31	436	292	325	370	617	436
9	01011000	51	84	24	84	99	31	416	312	325	370	637	416
10	00111000	51	39	86	84	99	31	399	329	325	370	654	399
11	11000100	76	84	24	37	34	98	436	290	327	370	617	436
12	00100100	51	39	86	37	34	98	444	282	327	370	609	444
13	10100100	76	39	86	37	34	98	419	307	327	370	634	419
14	01100100	51	84	86	37	34	98	399	327	327	370	654	399
15	10010100	76	39	24	84	34	98	434	292	327	370	619	434
16	01010100	51	84	24	84	34	98	414	312	327	370	639	414
17	00110100	51	39	86	84	34	98	397	329	327	370	656	397
18	00001100	51	39	24	37	99	98	441	220	392	370	612	441
19	10001100	76	39	24	37	99	98	416	245	392	370	637	416
20	01001100	51	84	24	37	99	98	396	265	392	370	657	396
21	00101100	51	39	86	37	99	98	379	282	392	370	674	379
22	00011100	51	39	24	84	99	98	394	267	392	370	659	394

**Table 2:** Values of objective functions

Feasible plans	1	2	3	4	5	6	7	8	9	10	11	12	13	14	15	16	17	18	19	20	21	22
*λ* 1	7	7	7	7	5	7	7	7	7	7	7	5	7	7	7	7	7	5	7	7	7	7
*λ* 2	441	439	419	438	446	421	401	436	416	399	436	444	419	399	434	414	397	441	416	396	392	394
*λ* 3	86	86	86	99	99	99	99	99	99	99	98	98	98	98	96	98	98	99	99	99	99	99
*λ* 4	38	37.8	36	36	36.7	31.7	28.6	34.6	30.9	28.4	34.7	36.4	31.4	28.4	34.3	30.6	28.1	44.3	38.5	34.4	31.5	34
*λ* 5	62.8	62.1	60.9	73.5	67	67.6	65.8	72.9	71.8	65	71.8	65.1	65.6	63.8	71.1	70	62.9	75.5	75.8	74.1	66.9	73.4
*λ* 6	441	439	419	615	607	632	652	617	637	654	617	609	634	654	619	639	656	612	637	657	674	659
*λ* 7	80.7	81.6	82.7	78.6	79.8	74.5	72.3	78.1	75.2	71.8	79.9	73.3	76.4	71.8	72.8	76.2	71.6	78.2	76.6	71.6	70.8	71.3

**Table 3:** Values of fuzzy evaluation data

Feasible plans	*λ* 1*	*λ* 2*	*λ* 3*	*λ* 4*	*λ* 5*	*λ* 6*	*λ* 7*
1	0.625	0.0391	0.3469	0.620	0.372	0.6315	0.305
2	0.625	0.0478	0.3469	0.622	0.379	0.6421	0.282
3	0.625	0.1348	0.3469	0.640	0.391	0.7777	0.204
4	0.625	0.0522	0.2143	0.650	0.266	0.4210	0.336
5	0.750	0.0174	0.2143	0.633	0.330	0.3226	0.325
6	0.625	0.1261	0.2143	0.683	0.324	0.1904	0.362
7	0.625	0.2130	0.2143	0.714	0.324	0.1666	0.481
8	0.625	0.0609	0.2143	0.654	0.271	0.1998	0.335
9	0.625	0.1478	0.2143	0.691	0.282	0.1876	0.354
10	0.625	0.2217	0.2143	0.716	0.350	0.1642	0.562
11	0.625	0.0609	0.2245	0.653	0.282	0.1998	0.325
12	0.750	0.0261	0.2245	0.636	0.349	0.1666	0.392
13	0.625	0.1348	0.2245	0.659	0.344	0.1898	0.354
14	0.625	0.2217	0.2245	0.716	0.362	0.1642	0.562
15	0.625	0.0696	0.2245	0.657	0.289	0.1878	0.480
16	0.625	0.1565	0.2245	0.694	0.300	0.1799	0.354
17	0.625	0.2304	0.2245	0.719	0.371	0.1582	0.562
18	0.750	0.0391	0.2143	0.557	0.245	0.2374	0.335
19	0.625	0.1478	0.2143	0.615	0.242	0.1876	0.354
20	0.625	0.2348	0.2143	0.656	0.259	0.1592	0.572
21	0.625	0.2522	0.2143	0.685	0.331	0.0833	0.625
22	0.625	0.2435	0.2143	0.667	0.266	0.1453	0.562

**Table 4:** Test result of grey relational grade

Feasible plan	GRG (old)	GRG (new)	Difference in GRG
1	0.612	0.6612	0.0492
2	0.6277	0.8304	0.2027
3	0.7263	0.8231	0.0968
4	0.4446	0.7221	0.2775
5	0.5624	0.9723	0.4099
6	0.5862	0.7869	0.2007
7	0.7011	0.6582	−0.0429
8	0.4611	0.8806	0.4195
9	0.576	0.6205	0.0445
10	0.718	0.4381	−0.2799
11	0.4779	0.6997	0.2218
12	0.5968	0.7461	0.1493
13	0.6212	0.576	−0.0452
14	0.7369	0.5327	−0.2042
15	0.4944	0.4994	0.005
16	0.6101	0.6113	0.0012
17	0.7539	0.6827	−0.0712
18	0.4435	0.7321	0.2886
19	0.4767	0.7261	0.2494
20	0.6001	0.8626	0.2625
21	0.7011	0.8251	0.124
22	0.6173	0.6621	0.0448

**Table 5:** Rank before and after restoration

Feasible plan	1	2	3	4	5	6	7	8	9	10	11
Grey relational grade	0.6612	0.8304	0.8231	0.7221	0.9723	0.7869	0.6582	0.8806	0.6205	0.4381	0.6997
Rank before fault	10	7	3	21	16	14	5	20	15	4	18
Rank after restoration	16	4	6	11	1	7	17	2	18	22	12

Feasible plan	12	13	14	15	16	17	18	19	20	21	22
Grey relational grade	0.7461	0.576	0.5327	0.4994	0.6113	0.6827	0.7321	0.7261	0.8626	0.8251	0.6621
Rank before fault	13	8	2	17	11	1	22	19	12	6	9
Rank after restoration	8	19	20	21	14	13	9	10	3	5	15

In this paper, the objective functions are considered so as to optimize the operation of switches and loading of feeders, laterals as well as on transformers and minimization of unbalanced loading index of feeders, laterals and transformer after switching operation. The constraints in the restoration process to be considered are: (a) maintain the radial structure of the network, (b) no overloaded equipment and (c) higher priority customers should always be supplied first. The result shows that inclusion of the transformer current limits has changed the ranking of plans. The system becomes more reliable with the minimization of unbalanced loading of transformer current as there is no scope for exceeding the limits during restoration plans. Feasible plans which consider more objective functions make the system more reliable. The reliability and stability of the restored network has increased by 1.09 %. The priority customers can be supplied first on the basis of the preference index plans during partial blackout or full blackout. The consumer loads which are not energized may be fed by the supporting feeders in the neighbourhood via on-off switches. The result shows that restoration process is done using minimum number of switching operations. Safety and operability of transformer, laterals and feeders is taken up by maintaining the line currents within the operational limits of power system components.

The topology of distribution system is maintained radial before and after implementing the restoration plans. A switch-opened operation is always followed by a switch-close operation, after every switching operation.

## Conclusion

Simulation studies for restoration of distribution system are carried out considering multi-objective problem and fuzzy-grey algorithm. Transformer loading has been considered as an additional objective function in the optimization problem. The result shown is more stable and reliable because more are the objective functions, greater is the stability. Various strategies are derived based on rank. The best strategy is the one with the highest rank. The studies are useful for system operator in taking right decision during the restoration process. The rank of restoration plans is given which makes this method effective and very promising. The studies are useful for electric utilities/power distribution company to improve the customer services and revenue returns.

## Discussion and future work

This research work has proposed an intelligent restoration algorithm for the distribution system using fuzzy grey combination. The computation implementation is done by adding the unbalance loading of transformer to the Taiwan Power Distribution network. The result shows that the stability of system has improved with the new additional objective functions. The fuzzy multicriteria evaluation gives the optimization values for various objective functions and using grey regression analysis the ranking of plans has been done. This can help in service restoration of priority based customers. The intelligent algorithm is capable of fulfilling the requirements of smart grid such as stability, automation and reliability. The power system automation enables rapid diagnosis and precise solutions to the particular network outages. The proposed algorithm can be useful in making the restoration process automatic with the predecided rank of plans.

## Appendix 1

See Table 6.

**Table 6:** Pre fault load current of laterals

Lateral	1	2	3	4	5	6	7	8	9	10	11	12	13	14	15	16	17
Current	25	45	62	47	65	67	60	55	20	51	39	24	37	34	31	60	80

## Appendix 2

See Table 7.

**Table 7:** Values of fuzzy function for objective function

S. no	Objective function	*λ*_*a*_	*λ*_*b*_	*λ*_*c*_
1	Number of switching operation	1	9	17
2	Loading among supported feeder (A)	220	345	450
3	Loading among supported laterals (A)	24	76	120
4	Unbalance loading index of feeders	0	50	100
5	Unbalance loading index of laterals	0	50	100
6	Loading among supported transformer (A)	320	500	680
7	Unbalance loading index of transformers	40	70	100
